# Selective Up-Regulation of Arginase-1 in Coronary Arteries of Diabetic Patients

**DOI:** 10.3389/fimmu.2013.00293

**Published:** 2013-09-26

**Authors:** Zsolt Bagi, Attila Feher, Huijuan Dou, Zuzana Broskova

**Affiliations:** ^1^Vascular Biology Center, Medical College of Georgia, Georgia Regents University, Augusta, GA, USA

**Keywords:** diabetes mellitus, nitric oxide, arginase, endothelium, coronary artery

## Abstract

Coronary artery disease (CAD) remains the leading cause of death in the Western societies. Diabetes mellitus (DM) is one of the highly prevalent diseases, which remarkably accelerates the development of CAD. Experimental evidence indicates that decreased bioavailability of coronary endothelial nitric oxide (NO) contributes to the development of CAD in DM. There are recent studies showing that a selective impairment of NO synthesis occurs in coronary arteries of DM patients, which is mainly due to the limited availability of endothelial NO synthase (eNOS) precursor, l-arginine. Importantly, these studies demonstrated that DM, independent of the presence of CAD, leads to selective up-regulation of arginase-1. Arginase-1 seems to play an important role in limiting l-arginine availability in the close proximity of eNOS in vessels of DM patients. This brief review examines recent clinical studies demonstrating the pathological role of vascular arginase-1 in human diabetes. Whether arginase-1, which is crucial in the synthesis of various fundamental polyamines in the body, will represent a potent therapeutic target for prevention of DM-associated CAD is still debated.

## Diabetes Leads to a Reduced Availability of NO in Coronary Arteries

Diabetes mellitus is associated with an increased incidence of cardiovascular diseases accounting for significant morbidity and mortality in the diabetic population. Coronary artery disease (CAD) is one of the key manifestations of diabetes-associated vascular disease, a pathology, which predisposes diabetic patients to myocardial ischemia. The underlying mechanism(s) of CAD remain incompletely understood in human diabetes, so that effective preventive therapeutic strategies cannot be adopted in diabetic patients.

The coronary flow-reserve, as defined by the ratio of coronary flow under maximal agonist-induced vasodilation to coronary flow under resting conditions, is reduced in diabetic patients, even in the absence of significant stenosis of epicardial coronary arteries (1). Nemes et al. have demonstrated that patients with type 2 diabetes exhibit a reduced coronary flow-reserve (2), a condition, which was found to be associated with increased incidence of future ischemic episode in the heart of diabetic patients (3). Patients with diabetes exhibit endothelial dysfunction, which is characterized by impaired flow- and acetylcholine (ACh)-induced relaxation of brachial artery (4) and forearm resistance vessels (5). Nitenberg et al. have demonstrated that coronary artery dilation is impaired in diabetic patients with angiographically normal coronaries (6). Kaneda et al. performed a study, in which 165 patients underwent intra-coronary injection of ACh and found that diabetes was the strongest predictor for ACh-induced coronary vasospasm (7). This and other studies concluded that diabetes is associated with impaired dilator function of coronary arteries and this is manifested as a reduced vasodilator or even vasoconstrictor responses (8–10).

Previous studies have shown that animals with experimental insulin resistance and diabetes exhibit a reduced NO-mediated, agonist-induced dilation of cerebral, mesenteric, coronary, and skeletal muscle microvessels (11–16). Studies from our laboratory demonstrated that in rodent models of type 2 diabetes coronary arteries exhibit impaired ACh-induced dilation, which is primarily due to the reduced synthesis and/or bioavailability of nitric oxide (NO) (17–20). Katakam et al. have shown that prior to the impaired ACh-mediated vasodilation NO-mediated coronary dilation to insulin is reduced in obese Zucker rats (14). This seems particularly important as insulin and insulin-like growth factor I have shown to promote NO-mediated vasodilation (21).

Oxidative stress occurring in response to hyperglycemia and insulin resistance (14, 22–28) is considered to be one of the key factors leading to the reduced NO-dependent vasodilation. To support this scenario, oral administration of the antioxidant vitamin-C prevented the decreases in methacholine-induced brachial artery dilations in patients with diabetes (29). However, other studies failed to detect any beneficial effect of antioxidant therapy in the prevention of diabetes-induced vascular complications (30, 31). For instance, vitamin-E supplementation for 8 weeks did not restore the reduced ACh- and bradykinin-induced dilations of brachial arteries in diabetic patients (32). These aforementioned observations raised questions about the efficacy of antioxidant therapy in preventing diabetes-related endothelial dysfunction. To solve the apparent controversy recent studies propose a crucial role for reactive nitrogen species in the development of diabetes-related vascular complications (33). The rate constant for the reaction between superoxide anion and NO is three to fivefold greater than the rate of superoxide anion scavenged by superoxide dismutase (34). Given that NO via interacting with superoxide anion generates various reactive nitrogen species, such as the highly reactive peroxynitrite (ONOO^−^). ONOO^−^ has numerous detrimental effects in the cardiovascular system and plays a crucial role in the development of diabetes-induced vascular pathology (33). ONOO^−^ is a powerful oxidizing agent that causes rapid depletion of sulfhydryl groups, causes DNA damage, protein oxidation, and nitration of aromatic amino acid residues in proteins, specifically leading to 3-nitrotyrosin formation (33). Although the endogenous cellular mechanisms to prevent the deleterious effect of ONOO^−^ are not clearly defined recent preclinical studies suggest that a more selective targeting of ONOO^−^ holds considerable potential than the use of conventional antioxidants (35).

## Deficiency of eNOS Cofactors in Coronary Artery Disease

It is known that an adequate level of substrates and cofactors for NO synthases, such as l-arginine (36) and tetrahydrobiopterin (BH_4_) is essential for NO synthesis (37, 38). Diabetes has been shown to interfere with the availability of these cofactors thereby leading to a diminished NO synthesis. To provide experimental evidence for this scenario Ihlemann et al. demonstrated that in healthy humans, oral glucose challenge-induced reduction in forearm blood flow is restored by pre-treatment with BH_4_ (39). Co-infusion of BH_4_ and the endothelial NO synthase (eNOS) precursor, l-arginine into the forearm of diabetic patients prevented ischemia reperfusion-induced endothelial dysfunction in the brachial artery (40). In isolated coronary arterioles of patients with atherosclerosis Tiefenbacher et al. has shown earlier that *in vitro* administration of the stable BH_4_ analog, sepiapterin enhanced dilation in response to agonist (41). In a recent study oral BH_4_ treatment in patients undergoing heart surgery although augmented total biopterin levels had no significant effects on dilator function of conduit vessels owing to systemic and vascular oxidation of BH_4_ (42). This latter study warranted the need of further investigations aiming at effectively restoring eNOS cofactor, BH_4_ levels. Underlying mechanism(s) responsible for the reduced vascular availability of BH_4_ is not entirely understood in diabetes. It has been shown that ONOO^−^ directly interacts and reduces the level of BH_4_, as it has greater affinity for BH_4_ than that of ascorbic acid and glutathione (43). Chen et al. demonstrated that exposure of human eNOS to ONOO^−^ resulted in a dose-dependent loss of activity with a marked destabilization of the eNOS dimer (44). In addition, Ishii et al. has shown that insulin is a potential stimulator of BH_4_, primarily via activating phosphatidylinositol 3-kinase (45). Due to the apparent lack of insulin action expression and activity of GTP cyclohydrolase-I is reduced in insulin resistance states, which ultimately leads to eNOS uncoupling and diminished NO-mediated dilation of cerebral arteries (46). Whether restoring insulin sensitivity will be associated with “re-coupling” of eNOS has yet to be elucidated.

## l-Arginine to Prevent Endothelial Dysfunction in Patients with CAD

l-arginine, the substrate for NO synthase, is the precursor for NO synthesis in the vascular endothelium. Earlier clinical studies indicated that administration of l-arginine may enhance NO bioavailability and dilate coronary arteries (47). For example, intra-coronary infusion of l-arginine in patients with CAD attenuated the vasoconstrictor response to intra-coronary ACh and increased coronary blood flow (48). Lerman et al. studied the effect of long-term administration of l-arginine (9 g/day) on patients with non-obstructive coronary disease and found a markedly improved coronary vasodilator response to ACh (49). In contrast, in 30 patients with CAD l-arginine therapy while significantly increased l-arginine plasma levels had no effect on NO bioavailability and flow-mediated dilation of brachial artery (50). Moreover, there is another clinical study, which questioned the effectiveness of l-arginine therapy in patients with CAD (50). More importantly, in the Vascular Interaction With Age in Myocardial Infarction (VINTAGE MI) study by Schulman et al. l-arginine supplementation significantly increased mortality in patients with myocardial infraction leading to early termination of the trial with the recommendation that l-arginine supplementation not to be used in patients with myocardial infarction (51). The underlying mechanism responsible for the controversial and harmful effects of l-arginine in this particular patient population remains elusive.

Limited number of studies is available to evaluate the acute and long-term effects of l-arginine treatment in patients with diabetes. In a recent large cohort, involving 2236 patients recruited within the LUdwigshafen RIsk and Cardiovascular Health (LURIC) study, patients with type 2 diabetes had a significantly lower l-arginine availability than patients without diabetes (52). A study has shown that l-arginine treatment (8.3 g/day for 21 days) improved endothelial dilator function and increased insulin sensitivity in patients with type 2 diabetes (53). The authors concluded that l-arginine exerted its beneficial effects through reducing fasting and postprandial glucose levels and normalizing adiponectin/leptin ratio (53). Thus, some evidence indicates that in diabetic patients the level of l-arginine is reduced and administering l-arginine may improve endothelial function. Whether this effect is mediated directly via enhancing the vascular availability of NO or indirectly via increasing insulin sensitivity has yet to be elucidated. Taken together, it is possible that diabetic patients may benefit from l-arginine supplementation, but several important questions still remain open including the safety and efficacy of l-arginine treatment in diabetic patients with concomitant CAD, especially in those with prior myocardial infarction.

## Does Vascular Arginase-1 Selectively Up-Regulated in Human Diabetes?

It is known that the Km of NO synthase for l-arginine is about 2.9 μM. The intracellular concentration of l-arginine ranges from 0.1 to 1.0 mM. Given that it seemed intriguing how administration of l-arginine would increase the bioavailability of NO – the arginine-paradox. To solve this apparent controversy previous studies have shown that up-regulation of arginase, the focal enzyme of the urea cycle, via hydrolyzing l-arginine reduces NO synthesis and, importantly, may contribute to the development of various vascular diseases (36). Detailed pathological role(s) for arginase-1 and -2 in various disease models as well as the subcellular mechanisms behind is extensively discussed in other review articles in this thematic issue. In this review we examine those few existing clinical studies that focused on altered arginase expression and its vascular consequences in man.

Vascular endothelial cells metabolize l-arginine mainly by arginase, which exists as two distinct isoforms, arginase-1 and -2. Arginase-1 is predominantly expressed in the liver and to a much lesser extent in other cell types, such as vascular endothelial cells, whereas expression of arginase-2 is more widespread (54). Circulating arginase-1 level was found significantly higher in patients with heart failure, when compared to controls; and the level of circulating arginase-1 further increased with the severity of heart failure (significant increase between NYHA I/II and NYHA III/IV groups) (55). When sublingual microcirculation was assessed by dark field intravital microscopy in heart failure patients the authors found that topical administration of the arginase inhibitor, nor-NOHA increased capillary density, a measure of tissue perfusion, in an NO-dependent manner (55). In patients with essential hypertension Holowatz and Kenney have found an attenuated NO-dependent reflex cutaneous vasodilatation, which is enhanced by arginase inhibitors, BEC, and nor-NOHA, but not with l-arginine supplementation (56). These studies provided functional evidence for the role of circulating and also tissue-expressed arginase-1, which interferes with NO-mediated tissue perfusion.

Both arginase-1 and -2 are expressed in the human heart (54). In a study by Chen et al. right atrial appendage was obtained from 13 patients undergoing heart surgery for coronary artery bypass graft (CABG) with three-vessel CAD and from 13 patients with valve replacement (non-CAD group). In the whole atrial homogenates there was a significantly reduced protein expression of both eNOS and arginase-1 in patients with CAD. Of note that in this study the CAD group had higher proportion of diabetic patients (11 out of 13), as compared to the control group (3 out of 13). Given that, the individual impact of CAD and diabetes affecting expression of arginase-1 cannot be examined in this study (57). In our recent study (10) small coronary arteries were dissected from the atrial appendage of 21 patients without and 20 patients with diabetes. Protein expression of arginase-1 in small coronary arteries was significantly higher in patients with diabetes. Arginase-1 expression was abundant in endothelial cells and was co-localized with eNOS in coronary vessels of diabetic patients, but not in non-diabetics. We demonstrated that inhibition of arginase, with l-NOHA caused restoration of endothelium-dependent, ACh-induced dilation in coronary arterioles of diabetic patients. Although we proposed an effect of diabetes causing arginase-1 up-regulation, it is of note that in the study diabetic patients exhibited a significantly greater proportion of CAD (16 out of 20 versus 9 out of 20 patients), therefore the conclusion regarding the selective up-regulation of arginase-1 in coronary arteries of diabetic patients is severely limited. In a very recent study by Shemyakin et al. the independent impact of diabetes in affecting arginase-1 expression and its functional consequence was evaluated (58). In 16 patients with CAD and 16 patients with CAD and type 2 diabetes endothelium-dependent and endothelium-independent increases in forearm blood flow were assessed during intra-arterial infusion of the arginase inhibitor, nor-NOHA. While forearm blood flow was significantly lower in both CAD and CAD plus diabetes groups, when compared to age-matched control group, nor-NOHA markedly increased blood flow with NO-dependent manner, with a significantly greater extent in patients with concomitant diabetes (58). This key observation provided functional evidence for the selectively up-regulated arginase-1 in diabetic patients, *in vivo*.

Taken together, limited number of clinical studies suggest a selective up-regulation of arginase-1, which may impair dilator function of conduit and resistance vessels in diabetic patients, independent of the presence of concomitant CAD. Further studies involving higher number of research subjects are needed to assess the independent impact of increased arginase-1 expression on coronary artery responsiveness in diabetes. Moreover, it should be noted that arginase inhibitors, nor-NOHA, and l-NOHA used in these studies do not have selectivity toward arginase isoforms (arginase-1 versus arginase-2). Thus, the functional role for arginase-2 in the development of vasomotor dysfunction cannot be entirely excluded. In this regard, a previous study has found that increased expression of arginase-2 leads to decreased NO synthesis in pulmonary endothelial cells of patients with pulmonary arterial hypertension (59). The pathological role of arginase isoforms in diabetes-related coronary microvascular dysfunction also has yet to be elucidated.

## Possible Mechanisms Leading to Selective Up-Regulation of Arginase-1 in Diabetic Patients

**Figure 1:**
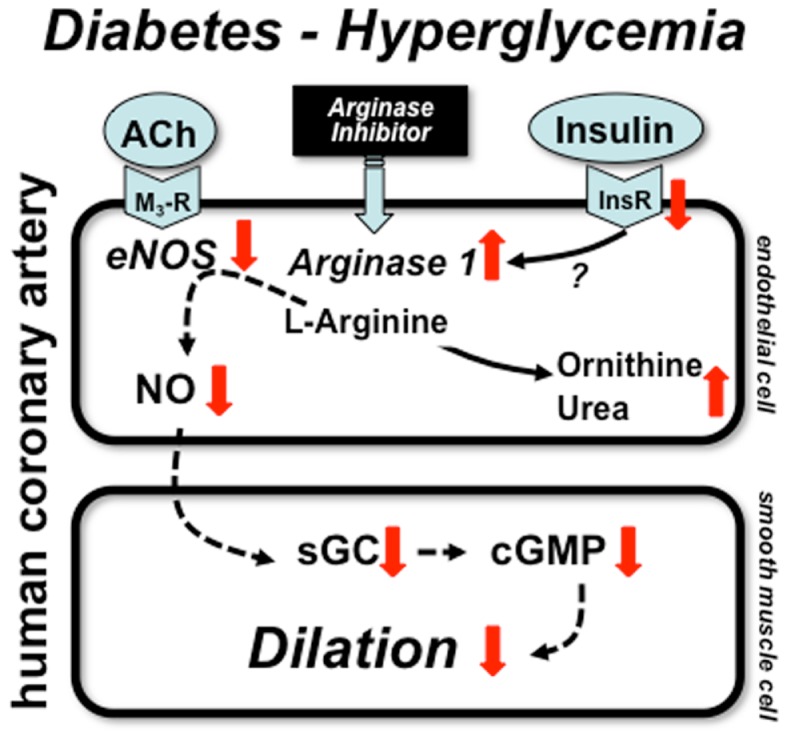
**Mechanisms of coronary artery dysfunction in diabetes**. Schematic draw demonstrates proposed mechanisms by which diabetes and hyperglycemia lead to up-regulation of arginase-1 resulting in reduced NO-mediated dilation in human coronary arteries. Alterations in insulin signaling may contribute to arginase up-regulation, which reduces the level of l-arginine and thereby limit NO synthesis. ACh: acetylcholine, M_3_-R: M_3_ muscarinic receptor, InsR: insulin receptor, sGC: soluble guanylate cyclase, cGMP: cyclic guanosine monophosphate.

The underlying mechanism(s) leading to selective up-regulation of arginase-1 in coronary arterioles in diabetic patients remains elusive. One obvious possibility is the known action of insulin, which suppresses expression and activity of enzymes of urea synthesis pathway. Since insulin signaling is impaired in type 2 diabetes (patients likely to exhibit insulin resistance) it is possible that the failure of insulin regulatory action contributes to up-regulation of arginase-1. In the clinical setting diabetic patients are on insulin sensitizing and oral anti-diabetic medication or commonly take insulin. For instance, in the study by Shemyakin et al. 31 and 56% of patients with CAD plus diabetes were on insulin and biguanides/sulfonylureas, respectively (58). In our aforementioned study all patients with diabetes had either anti-diabetic medication or were on insulin (10). Therefore, the effect of insulin resistance and the concomitant action of exogenous insulin is difficult to examine in these investigations. Also, due to the limited number of diabetic patients involved in the aforementioned studies further studies are warranted to ascertain the role of insulin and other pathological factors that could contribute to increased arginase-1 expression in diabetes. In this context, in a previous elegant study Kashyap et al. found that plasma arginase activity is increased in type 2 diabetic patients with reduced activity of eNOS in the skeletal muscle. Interestingly, these changes were detected without alterations in the plasma protein levels of arginase-1 and -2. Importantly, the increased arginase activation was correlated with the degree of hyperglycemia and was markedly reduced by 4-h insulin infusion in diabetic patients, but not in non-diabetics (60). This clinical study demonstrated the pathological role for high glucose in inducing, whereas for exogenous insulin in reducing arginase activation. The exact molecular mechanisms remained unclear. The proposed mechanisms by which diabetes and hyperglycemia lead to up-regulation of arginase-1 resulting in reduced NO-mediated dilation in human coronary arteries is depicted in Figure 1.

In summary, recent findings, as highlighted in this brief review underline the need for the investigations exploring the underlying mechanisms responsible for up-regulated arginase in human diabetes. Clinical studies emphasize the importance of those investigations that strive to elucidate the vascular effects of specific arginase inhibitors, including their long-term efficacy and safety in diabetic patients with CAD.

## Conflict of Interest Statement

The authors declare that the research was conducted in the absence of any commercial or financial relationships that could be construed as a potential conflict of interest.
